# Management of Emphysematous Cystitis in the Intensive Care Unit

**DOI:** 10.7759/cureus.111654

**Published:** 2026-06-28

**Authors:** Haroon Chaudhry, Ravali Kundeti, Madhvi Nagar, Muhammad Sameer Almas, Neveen Malik

**Affiliations:** 1 Critical Care Medicine, Thomas Jefferson University Hospital, Sewell, USA; 2 Medicine, Shri M. P. Shah Medical College, Jamnagar, IND; 3 Medicine, Allama Iqbal Medical College, Lahore, PAK; 4 Pulmonary and Critical Care Medicine, Thomas Jefferson University Hospital, Sewell, USA

**Keywords:** emphysema cystitis, emphysematous, gram-negative bacteremia, uncontrolled diabetes mellitus type 2, vasopressor support

## Abstract

Emphysematous cystitis (EC) is a complication of urinary tract infection (UTI) caused by gas-forming bacteria within the walls and lumen of the urinary bladder. We discuss here a case of a 55-year-old woman with uncontrolled diabetes mellitus, who received multiple courses of antibiotics for dysuria and who presented with altered mental status, hypotension, and hypoglycemia. Initially, the patient was managed with resuscitation, intravenous antibiotics, and supportive care. Eventually, she required vasopressors and was transferred to the Intensive Care Unit. Blood culture revealed* Proteus mirabilis.* CT abdomen and pelvis showed gas within the bladder wall along with new-onset left-sided pleural effusion. During the hospital stay, the patient’s vasopressor requirements escalated. She required invasive positive pressure ventilation and continuous venovenous hemodialysis due to worsened respiratory status and severe metabolic acidosis. The Urology Department determined that she was not a surgical candidate. Given her poor prognosis, the patient’s care was shifted to comfort measures only. This case highlights that EC in uncontrolled diabetics may present atypically with hypoglycemia rather than hyperglycemia, can be complicated by polymicrobial bacteremia and pleural effusion, and may progress to death despite maximal medical therapy. Clinicians should suspect EC in uncontrolled diabetics with hypoglycemia and septic shock. The presence of polymicrobial bacteremia or pleural effusion predicts a rapidly progressive and worse prognosis. When surgical intervention is not possible, mortality remains high despite aggressive medical management. Early urology consultation for possible cystectomy is essential before multiorgan failure ensues.

## Introduction

Emphysematous cystitis (EC) was first described by Eisenlohr [1] and Bailey [2]. It is a rare, life-threatening, complicated urinary tract infection (UTI) defined by gas within the bladder wall and lumen caused by gas-forming organisms. It predominantly affects middle-aged diabetic women and patients with neurogenic bladder, chronic UTIs, indwelling catheters, or urinary outlet obstruction. Presentation ranges from incidental imaging findings to severe sepsis and necrotizing cystitis. Prompt evaluation, early urology consultation, and aggressive management are essential [3]. The most common causative organisms are gram-negative bacteria, predominantly *Escherichia coli *and *Klebsiella pneumoniae *[4]. Diabetes mellitus is the strongest risk factor for EC, which presents in 50% to 70% of cases [3]. The pathogenesis of EC involves the fermentation of glucose (particularly in diabetics) or protein by the causative organisms within the affected tissues. The clinical presentation of EC is highly variable, from asymptomatic incidental finding to severe sepsis; therefore, it requires prompt evaluation, early diagnosis, and management [5]. Here we present a case of a middle-aged female with uncontrolled diabetes who developed gram-negative bacteremia and EC.

## Case presentation

A 55-year-old woman with a past medical history of uncontrolled type 2 diabetes mellitus, liver cirrhosis secondary to hepatic steatosis and hepatitis B, and morbid obesity (BMI 35-35.9 kg/m2) was brought to the emergency department (ED) due to lethargy, altered mental status, hypoglycemia, hypoxia, and hypotension. The patient had reportedly received multiple courses of antibiotics over the past three months for dysuria. Additional comorbidities included hyperlipidemia, hypertriglyceridemia, schizoaffective disorder, transient ischemic attack, migraine, and cervical stenosis.

On arrival at the emergency department, the patient appeared acutely ill and toxic. She was tachycardic with sinus rhythm and exhibited increased work of breathing, with diffuse wheezing noted bilaterally on lung auscultation. Physical examination revealed marked bilateral +4 pretibial edema with generalized anasarca. The abdominal examination was negative for any findings. Neurologically, she was arousable only to noxious stimuli and remained confused.

Initial point-of-care glucose was 48 mg/dL, reflecting her uncontrolled diabetes mellitus with depleted glycogen stores, high consumption of glucose by tissue, and possibly impaired counter-regulatory hormone response. It is a marker of poor organ perfusion. Hypoglycemia was promptly corrected with intravenous dextrose.

Further laboratory evaluation revealed profound acidemia with anion gap metabolic acidosis; arterial blood gas demonstrated a pH of 7.20, partial pressure of carbon dioxide (pCO₂) of 24 mmHg, and bicarbonate of 13 mmol/L, with serum bicarbonate of 10 mmol/L, consistent with a severe high anion gap metabolic acidosis with appropriate respiratory compensation. Lactate levels were markedly elevated and worsening (7.5 mmol/L rising to 9 mmol/L), consistent with septic shock.

Inflammatory markers were markedly elevated, with C-reactive protein at 15.4 mg/dL and procalcitonin at 5.49 ng/mL, confirming severe bacterial infection. Hypoalbuminemia (1.2 g/dL) in the context of liver cirrhosis reflected hepatic failure. Acute kidney injury was evidenced by creatinine of 5.68 mg/dL (baseline 0.85 mg/dL) and blood urea nitrogen (BUN) of 48 mg/dL, with an estimated glomerular filtration rate (eGFR) of 8, representing severe acute kidney injury (AKI).

Urinalysis demonstrated blood 3+, leukocytes 3+, hyaline casts of 309/LPF, RBC >182, and WBC >182, consistent with both upper and lower urinary tract involvement and intrinsic renal parenchymal injury. Blood cultures grew *Proteus mirabilis*, confirming Gram-negative bacteremia as the source of systemic sepsis.

Admission laboratory values are given in Table 1.

**Table 1:** Admission Laboratory Values

Investigation	Results	Reference Range
Sodium (Na+)	134 mEq/L	136-145.5 mEq/L
Potassium (K+)	4.9 mEq/L	3.6-5.1 mEq/L
Chloride (Cl-)	109 mEq/L	95-105 mEq/L
Bicarbonate (HCO3-)	10 mEq/L	23.1-29.5 mEq/L
Anion Gap	15 mmol/L	4-12 mmol/L
Blood Urea Nitrogen (BUN)	48 mg/dL	6-24 mg/dL
Creatinine (Cr)	5.68 (Baseline 0.85) mg/dL	0.7-1.3 mg/dL
BUN/Cr Ratio	8.4 mg/dL	10/1-20/1
Glucose	155 mg/dL	70-100 mg/dL
Alanine Aminotransferase (ALT)	40 U/L	8-33 U/L
Aspartate Aminotransferase (AST)	69 U/L	6.9-55.9 U/L
Protein, Total	3.8 g/dL	5.9-8.1 g/dL
Albumin	1.2 g/dL	3.0-5.2 g/dL
Direct, Bilirubin	0.4 mg/dL	0.1-1.2 mg/dL
Phosphate	1.82 mg/dL	2.8-4.3 mg/dL
Magnesium	1.3 mEq/L	1.2-2.0 mEq/L
Calcium	6.1 mg/dL	8.5-10.2 mg/dL
Lactic Acid	9 mmol/L	0.5-2 mmol/L
Estimated Glomerular Filtration Rate (eGFR)	8	>60
pH	7.20 mmHg	7.35-7.45 mmHg
Partial Pressure of Carbon Dioxide (pCO2)	24 mmHg	35-48 mmHg
Partial Pressure of Oxygen (pO2)	230 mmHg	83-108 mmHg
Prothrombin Time	>100 sec	9.4-13 sec
Partial Thromboplastin Time	87 sec	25-37 sec
International Normalization Ratio	>8.59	0.87-1.19
Procalcitonin	5.49 ng/ml	<0.10 ng/ml
White Blood Cell Count	15.9 x10^9/L	4.5-11 x10^9/L
Hemoglobin	9.3 g/dL	13.8-17.2 g/dL
Platelets	149 x10^9/L	150-400 x10^9/L
Creatine Kinase	675 IU/L	59-135 IU/L
Hepatitis B Core Antibody	Positive	Negative
D-Dimer	385 ng/mL	<242 ng/mL
Fibrinogen	242 mg/dL	200-393 mg/dL

In the ED, the patient received 1 liter of normal saline as initial resuscitation. A full 30 cc/kg septic bolus was withheld due to anasarca, which indicated severe total body fluid overload despite intravascular depletion. Aggressive fluid resuscitation in this case could have resulted in worsening pulmonary edema and tissue perfusion instead of improving it. Albumin 25 grams IV was also administered in the setting of hypoalbuminemia in order to maintain the plasma oncotic pressure and expand intravascular volume. Within an hour of admission, broad-spectrum antibiotics were initiated with piperacillin-tazobactam (4.5 g IV every 6 hours) and vancomycin (renally dosed targeting trough levels of 15-20 mg/L, administered every 12 hours with levels checked prior to the fourth dose). Stress-dose steroids were started given the high risk of adrenal decompensation in the setting of multiple comorbidities, hypoxia, and refractory septic shock. Resuscitation included continuous cardiopulmonary monitoring, IV fluid administration, blood culture collection, and serial lactate measurement. A Foley catheter was placed for bladder decompression and urine output monitoring, and vasopressor support was initiated for persistent hypotension. Despite these measures, the patient failed to improve over the subsequent four to five hours and was transferred to the intensive care unit. Shortly thereafter, her mentation modestly improved. Glasgow Coma Scale (GCS) score on arrival in the ED was 9, eventually improving to 11. She became slightly more awake and was able to answer basic questions. She was maintained on 6 liters of supplemental oxygen via nasal cannula with an oxygen saturation of 99%. Vasopressor requirements decreased, with norepinephrine 0.3 mcg/kg/min via a peripheral IV. Bedside ultrasound demonstrated a hyperdynamic left ventricular function and an inferior vena cava measuring approximately 1 cm with 50-75% inspiratory collapse, suggesting fluid responsiveness.

Computed tomography of the abdomen and pelvis revealed hepatic steatosis and extensive gas within the bladder wall, consistent with emphysematous cystitis (Figures 1, 2, 3). Additional findings included new multifocal bronchocentric and subpleural ground-glass opacities in both lungs, suggestive of infectious or inflammatory etiology, as well as a new moderate left-sided pleural effusion.

**Figure 1:**
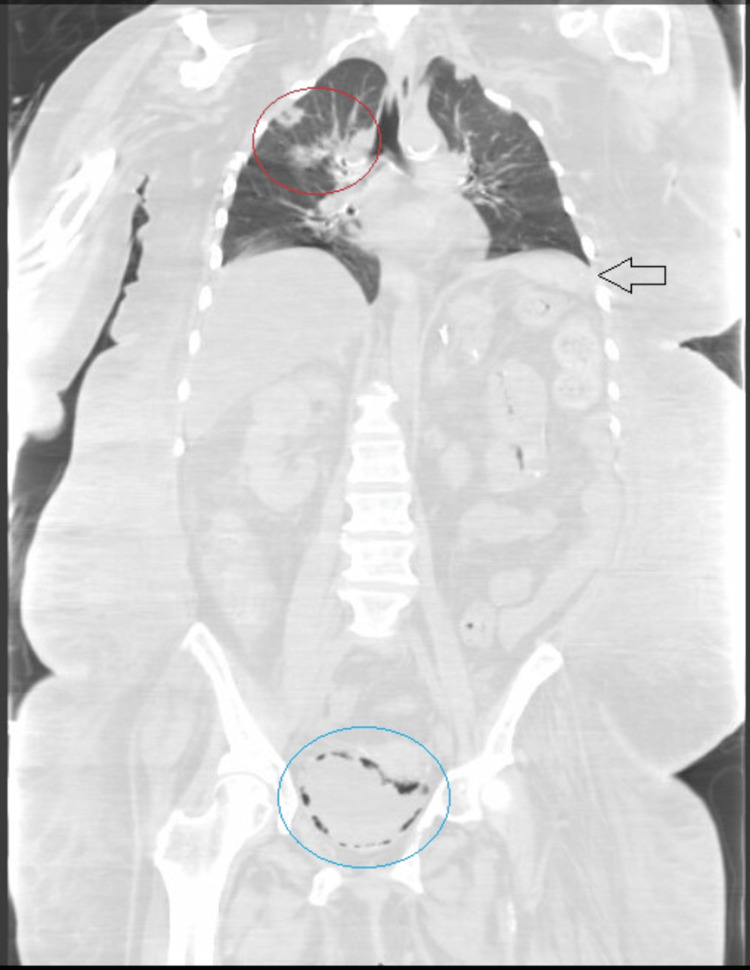
Coronal CT of the Chest, Abdomen, and Pelvis Coronal CT of the chest, abdomen, and pelvis demonstrating extensive intramural and intraluminal gas within the bladder wall (blue circle), consistent with emphysematous cystitis. Incidental findings include left-sided pleural effusion (black arrow) and bilateral ground-glass opacities (red circle), reflecting concurrent pulmonary involvement.

**Figure 2:**
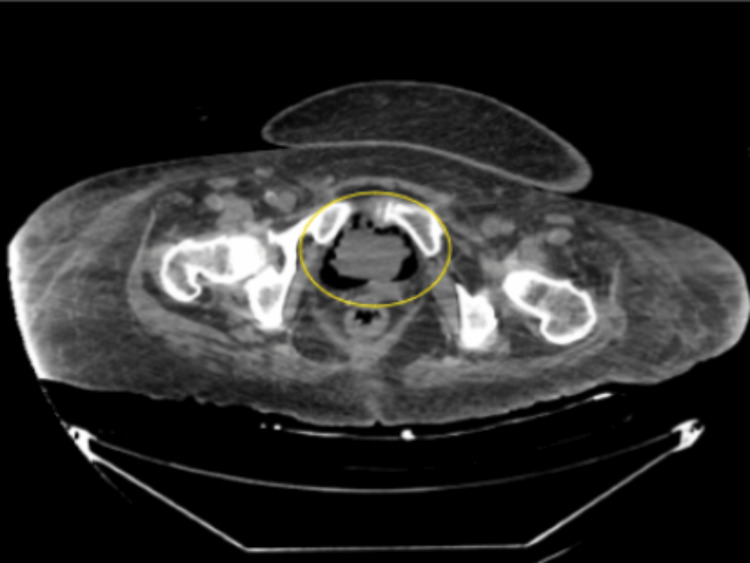
Axial CT of the Abdomen and Pelvis. Axial CT of the abdomen and pelvis demonstrating the classic "beaded necklace" pattern of intramural gas within the bladder wall (yellow circle), pathognomonic for emphysematous cystitis.

**Figure 3:**
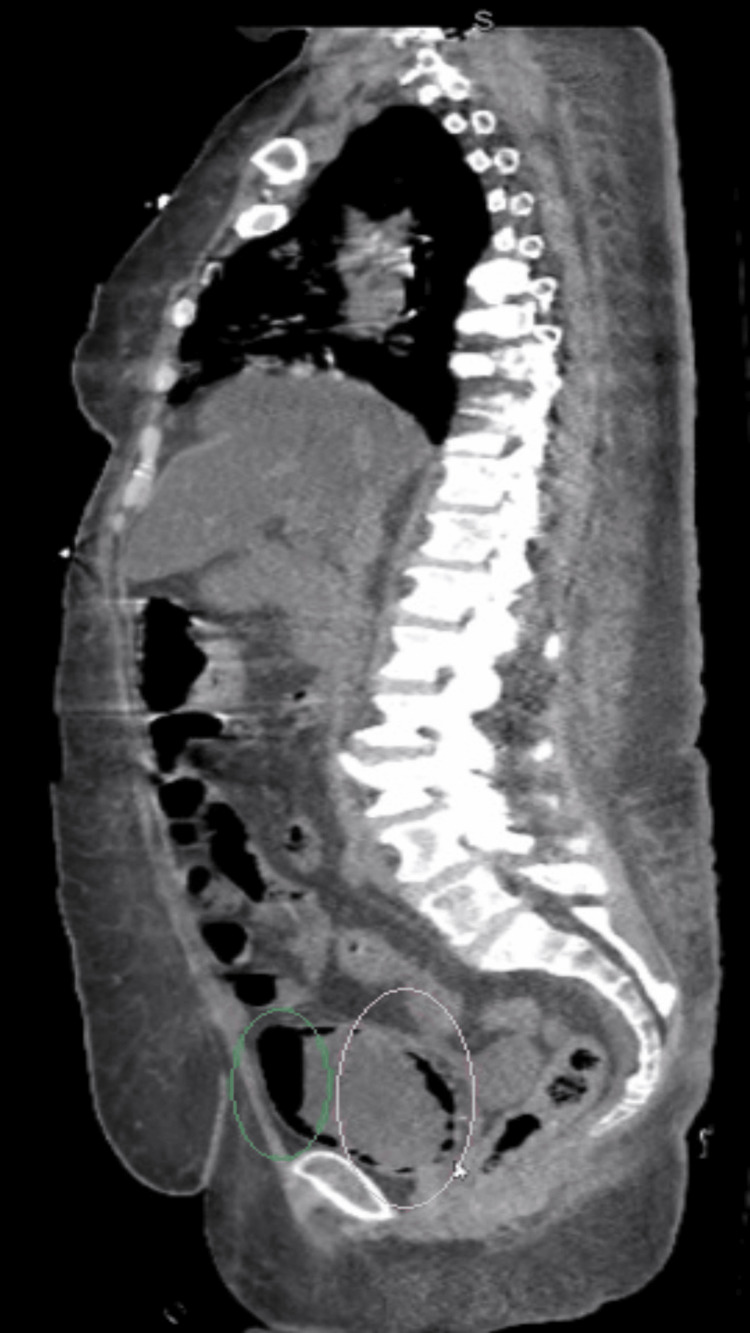
Sagittal CT of the Abdomen and Pelvis. Sagittal CT of the abdomen and pelvis demonstrating intraluminal gas forming an anterior air column (green circle) and intramural gas in the characteristic beaded necklace distribution along the bladder wall (pink circle), confirming both luminal and mural involvement in emphysematous cystitis.

Urology was consulted for gas in the bladder (Figure 3) and recommended Foley catheter placement to achieve maximal bladder decompression. EC can be managed surgically via total or partial cystectomy or drainage if medical interventions fail. It was not feasible in this case due to uncorrectable coagulopathy (international normalized ratio (INR) >8.59, partial prothrombin time (PTT) >87 sec), severe thrombocytopenia (149 K/μL), and hemodynamic instability requiring four vasopressors. In retrospect, earlier CT imaging and urology consultation might have allowed surgical intervention before the coagulopathy developed.

During the hospital course, the patient developed worsening respiratory status and required endotracheal intubation and mechanical ventilation for airway protection on hospital day 1. Later on, she progressed to severe metabolic acidosis and rapidly escalating vasopressor requirements, eventually needing four vasopressors (all at maximum doses). She developed worsening acute kidney injury, necessitating initiation of continuous venovenous hemodialysis (CVVHD). In cirrhotic patients with sepsis, the acute kidney injury in this patient represents a nearly sevenfold rise in creatinine from baseline, which warrants specific consideration in the context of her underlying cirrhosis. In cirrhotic patients with sepsis, AKI is usually one of two types: acute tubular necrosis (ATN), caused by reduced blood flow or toxins, or hepatorenal syndrome (HRS-1), a functional kidney failure caused by severe blood vessel dilation in the abdomen. The urinalysis in this patient demonstrated abundant hyaline casts, frank hematuria, and pyuria alongside a low BUN/creatinine ratio and profound hemodynamic compromise with lactate of 9 mmol/L. These findings are most consistent with ischemic ATN as the predominant mechanism. However, given the severity of underlying cirrhosis and hypoalbuminemia, a superimposed component of HRS-1 cannot be excluded, particularly as the two processes frequently coexist in septic cirrhotic patients. Her renal function did not recover despite resuscitation and dialysis, suggesting a mixed form that was particularly refractory to treatment. Given her critical condition and poor prognosis, goals of care were discussed with the family, and the patient was transitioned to comfort-focused measures.

This case highlights several key takeaways. Hypoglycemia in a diabetic septic patient must not be dismissed, as it may represent an early sign of metabolic decompensation; in our case, it was the first clue to an underlying gas-forming infection. Second, EC can cause fulminant clinical deterioration. This patient progressed rapidly to shock, respiratory failure, and refractory acidosis within hours of presentation. Third, underlying cirrhosis depleted the coagulation reserve, making hemorrhagic complications uncorrectable. Non-operative management comprising antibiotics, Foley catheter drainage, and glycemic optimization remains the first-line approach. However, this case demonstrates that severe EC complicated by multiorgan failure and coagulopathy can be fatal despite adequate medical management.

## Discussion

Emphysematous cystitis (EC) is a rare gas-forming infection of the bladder wall and lumen, most commonly affecting elderly women with poorly controlled diabetes mellitus. The median age at presentation is approximately 66 years, and the condition occurs twice as frequently in women as in men. Escherichia coli accounts for the majority of cases (approximately 55%), followed by Klebsiella pneumoniae (approximately 21%) [5,6]. Less common causes include Pseudomonas aeruginosa, Proteus mirabilis, Streptococcus species, and Candida species. Rarely, Aspergillus fumigatus has been reported in cases [7,8] Beyond diabetes, neurogenic bladder, urinary tract obstruction, indwelling urethral catheters, recurrent urinary tract infections, and immunodeficiency also contribute as predisposing factors [9]. This patient carried several of these risk factors simultaneously: poorly controlled diabetes and a history of recurrent urinary tract infections. This patient received multiple courses of antibiotic regimens over 3 months. Such patients can develop multidrug-resistant organisms, which can contribute to worse outcomes.

The main risk factor for EC is uncontrolled diabetes mellitus with the presence of significant glucosuria. Gas-forming organisms ferment intravesical glucose under hypoxic conditions, producing carbon dioxide and hydrogen, which collect in the lumen and bladder walls. In non-diabetic patients, organisms may utilize tissue lactate or albumin as fermentation substrates [10]. Additional diabetes-related mechanisms that increase the risk of EC include bladder dysfunction from peripheral neuropathy, impaired leukocyte function, and renal papillary necrosis [5]. In the present case, hypoalbuminemia (albumin 1.2 g/dL) in the setting of liver cirrhosis contributed to poor perfusion and delayed infection control [11].

The clinical presentation of EC is highly variable, ranging from incidental asymptomatic detection on imaging, accounting for up to 7% of cases, to severe sepsis with multiorgan failure [5]. Common presenting symptoms include fever, abdominal or suprapubic pain, dysuria, urinary frequency, and urgency. Pneumaturia, though highly specific, is uncommonly reported by patients. There are no significant clinical features that strongly suggest the diagnosis and distinguish it from uncomplicated urinary tract infection, underscoring the importance of imaging in any deteriorating patient with risk factors for gas-forming infection. In this case, the initial presentation was of classic urinary symptoms along with altered mentation and hypoglycemia. The latter, in a diabetic patient presenting with sepsis, should not be attributed to insulin excess. Hypoglycemia in the context of sepsis reflects hepatic failure of gluconeogenesis and increased peripheral glucose consumption, a marker of metabolic decompensation [12].

Imaging is not usually performed in UTI cases. However, EC can lead to bladder rupture, septicemia, peritonitis, and subcutaneous emphysema, with a mortality rate of 7%. When EC presents with emphysematous lesions of other organs (such as emphysematous pyelonephritis), the mortality rate increases to 14-20%. Hence, every severely ill diabetic patient with a urinary tract infection (especially presenting with septic shock requiring escalating vasopressor doses) should undergo extensive imaging. An abdominal radiograph can be helpful sometimes to screen for emphysematous complications, showing rims of gas lucency in the bladder wall and/or lumen. Elevated gas content seen in extra-urologic structures such as the vagina or uterus can obscure the diagnosis [13,14].

Computed tomography of the abdomen and pelvis is the investigation of choice, offering high sensitivity for detecting intraluminal and intramural gas. It helps in identifying disease extent and differentiating EC from other gas-forming conditions [15]. This distinction is critical, as concurrent upper tract involvement carries a mortality of 14-20% compared with approximately 7% for isolated EC [3,16]. The classic CT appearance is a beaded pattern of gas within the bladder wall or air-fluid levels within the lumen, as seen in this case [17]. Plain radiography may suggest the diagnosis but lacks sensitivity. Urine and blood cultures are needed for pathogen identification and antibiotic optimization.

Medical management, involving broad-spectrum antibiotics, Foley catheter drainage, and glycemic control, is successful in approximately 90% of cases [3]. Surgical intervention includes bladder lavage, cystotomy, partial cystectomy, or total cystectomy; it is reserved for the approximately 10% of patients who fail to respond to medical therapy or those with bladder perforation, extravesical abscess, or concomitant emphysematous pyelonephritis [18]. In this patient, surgical management was precluded by uncorrectable coagulopathy (INR >8.59, PTT >87 seconds), severe thrombocytopenia, and hemodynamic instability requiring four vasopressors at maximal doses. In retrospect, earlier cross-sectional imaging and urological consultation may have identified a window for surgical intervention prior to the development of irreversible coagulopathy.

This case demonstrates how EC, though treatable in the majority of patients, can follow a fatal course when comorbidities like cirrhosis, hypoalbuminemia, and prior antibiotic exposure converge with delayed recognition. Patients presenting with shock on admission, altered mental status, and thrombocytopenia carry a mortality rate as high as 52.4%, far exceeding the 7% population-level figure, and this patient met all three criteria at presentation [3,19]. Hypoglycemia at presentation in a diabetic patient with sepsis should not be attributed to insulin excess; it may represent hepatic gluconeogenic failure and serve as an early marker of metabolic decompensation [12]. Prompt CT imaging, early source control, and multidisciplinary involvement are necessary for management in high-risk patients with EC.

## Conclusions

Emphysematous cystitis is an uncommon UTI caused by gas-forming bacteria, occurring most often in patients with uncontrolled diabetes mellitus. We presented a case of a female patient with uncontrolled diabetes mellitus and long-term urinary symptoms, which demonstrates the importance of timely diagnosis and management to prevent serious complications and achieve a favorable prognosis. It commonly presents with septic shock, sometimes requiring adequate fluid resuscitation and vasopressor support. EC should be suspected in uncontrolled diabetic patients with sepsis, even if the presenting complaint is hypoglycemia. CT imaging of the abdomen and pelvis is necessary in such patients for early diagnosis. Clinicians must be on the lookout for alarm signs like pleural effusion, coagulopathy, and the presence of polymicrobial bacteremia. Urgent urological consultation and surgical intervention are needed before Disseminated Intravascular Coagulation (DIC) or hepatic failure ensues.
